# New titania-based photocatalysts for hydrogen production from aqueous-alcoholic solutions of methylene blue

**DOI:** 10.1039/d0ra07630a

**Published:** 2020-09-15

**Authors:** Dina V. Markovskaya, Angelina V. Zhurenok, Anna Yu. Kurenkova, Anna M. Kremneva, Andrey A. Saraev, Sergey M. Zharkov, Ekaterina A. Kozlova, Vasily V. Kaichev

**Affiliations:** a Federal Research Center Boreskov Institute of Catalysis Lavrentiev Ave., 5 Novosibirsk 630090 Russia kozlova@catalysis.ru; b Kirensky Institute of Physics, Federal Research Center KSC SB RAS Akademgorodok 50/38 Krasnoyarsk 660036 Russia; c Siberian Federal University 79 Svobodny pr. Krasnoyarsk 660041 Russia

## Abstract

A series of CuO_*x*_–TiO_2_ photocatalysts were prepared using fresh and thermally activated Evonik Aeroxide P25 titanium dioxide. The photocatalysts were characterized by X-ray diffraction, transmission electron microscopy, X-ray photoelectron spectroscopy, XANES, diffuse reflectance spectroscopy, and N_2_ adsorption technique. Photocatalytic activity of the samples was tested in hydrogen production from aqueous-alcoholic solutions of methylene blue under UV radiation (*λ* = 386 nm). It was found for the first time the synergistic effect of hydrogen production from two substrates—dye and ethanol. The maximum hydrogen production rate in the system water–ethanol–methylene blue was 1 μmol min^−1^, which is 25 times higher than a value measured in a 10% solution of ethanol in water. The thermal activation of titania also leads to a change in the rate of hydrogen production. The highest catalytic activity was observed for a CuO_*x*_–TiO_2_ photocatalyst based on titania thermally-activated at 600 °C in air. A mechanism of the photocatalytic reaction is discussed.

## Abstract 2

Simultaneous presence of ethanol and methylene blue was shown to provide the most efficient hydrogen production and methylene blue removal.

## Graphical abstract

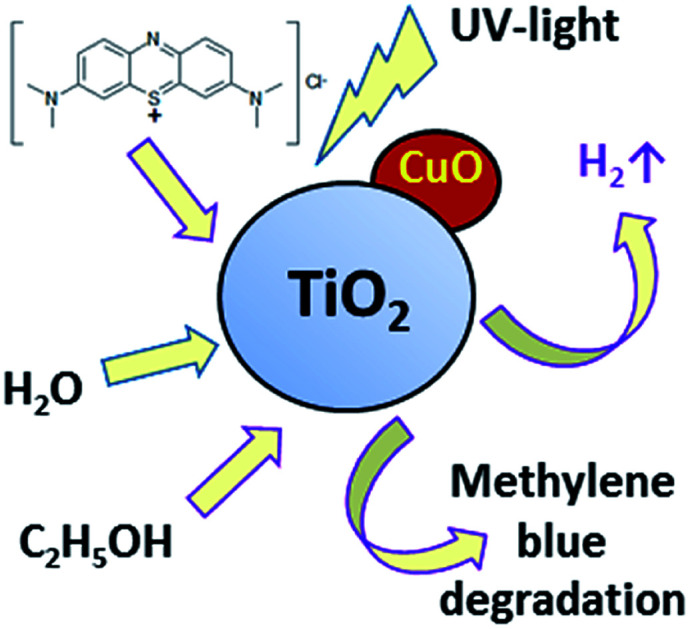

## 1 Introduction

Currently, photocatalytic processes, including hydrogen production under the influence of light, are recognized as one of the most important sustainable energy processes. Of particular interest is the photocatalytic production of hydrogen from aqueous solutions of various organic substances, electron donors, which can be water pollutants. In this case, two important processes – hydrogen production and water purification – can be combined.^1^ Organic dyes can be considered promising electron donors for hydrogen production.

Methylene blue, which is an organic thiazine dye, is applied in various fields, particularly, coloration of textiles,^2–4^ plastics and paper production,^2,3^ and serves as an antiseptic and a medicinal preparation.^4,5^ The dye is employed in analytical chemistry for determination of inorganic ions and some organic compounds^6^ and also as a redox indicator.^7^ The toxicity of methylene blue, which is consumed in large quantities, makes it necessary to develop effective methods of water purification from this dye.^2–6^ At present, water is purified using the adsorption methods,^8,9^ filtration,^2,3^ extraction,^2^ coagulation,^10^ and chemical oxidation and reduction of the dye.^11^ An essential drawback of the listed methods is low efficiency of water purification at low concentrations of the dye; at this stage, photocatalysis is considered to be a promising purification method. It is known that photocatalytic reactions allow decreasing the amount of organic impurities for a short time by their oxidation.^12^ A significant advantage of the photocatalytic method is the possibility of complete oxidation of the dye molecules to carbon dioxide and water.^3^ Among photocatalysts used for the removal of methylene blue from aqueous solutions, titania is the most popular one.^12^ This material is chemically stable, has an appropriate electrochemical potential for oxidation of the dye, and is not expensive.^13^ The effective approach to enhance the photocatalytic activity of the semiconductor material is the creation of heterojunctions.^14–16^ The rate and efficiency of photocatalytic purification can be enhanced with the use of various modifiers deposited on titania, particularly copper oxides.^17^ Also, thermal treatment of titania is known to improve its photocatalytic properties due to the changes of anatase/rutile ratio and surface properties.^18^ Recently, preliminary thermal treatment of titanium dioxide was used in our group to obtain Pt/TiO_2_ photocatalysts, which were active in the hydrogen production from glycerol aqueous solutions.^19^

It seems interesting to combine the photocatalytic oxidation of methylene blue with other processes. Earlier, it was demonstrated that the presence of titania allows oxidation of the dye and synthesis of hydrogen to occur simultaneously in seawater.^17^ However, the reaction rate of these processes was extremely low. In the present work, the process of photocatalytic hydrogen production was studied in aqueous and aqueous-alcoholic solutions of methylene blue in the presence of CuO_*x*_–TiO_2_ photocatalysts. Fresh and thermal activated commercial Evonik Aeroxide P25 titanium dioxide was used for preparation of the photocatalysts. The activation of titania was performed by calcination in air at a specified temperature in the range between 400 to 800 °C.

## 2 Materials and methods

### 2.1 Preparation of photocatalysts

A series of TiO_2_ P25-T*x* samples, where *x* is the calcination temperature, were obtained by heating the commercial TiO_2_ Evonik P25 for 3 h in air at a specified temperature in the range from 400 to 800 °C. After that, 1 wt% of copper was deposited on titania by impregnation with an aqueous solution of copper nitrate followed by reduction with a 2.5-fold excess of sodium borohydride. The photocatalysts were washed several times by decantation and dried at 50 °C for 4 h. The copper-modified photocatalysts were referred to as Cu-P25-T*x*, where *x* is the calcination temperature. For comparison, copper was deposited on the non-calcined titania, this sample was referred to as Cu-P25-RT.

### 2.2 Photocatalyst characterization

The phase composition of the photocatalysts was examined by X-ray diffraction (XRD) using a Bruker D8 Advance diffractometer equipped with a Lynxeye linear detector. The diffraction patterns were obtained in the 2*θ* range from 15° to 65° with a step of 0.05° using the monochromatic Cu Kα radiation (*λ* = 1.5418 Å). The phases were identified using the powder diffraction database PDF-4+. The mean sizes of crystallites in the samples were estimated as the coherent scattering region (CSR) from the full width at half maximum of corresponding peaks using the Scherrer equation. The quantitative content of phases was found by the Rietveld method using the TOPAS software.

The specific surface area (SSA) was calculated by the Brunauer–Emmett–Teller method using nitrogen adsorption isotherms measured at liquid nitrogen temperatures with an automatic Micromeritics ASAP 2400 sorptometer.

The microstructure and the local elemental composition of the samples were studied using a JEOL JEM-2100 transmission electron microscope (TEM) equipped with an Oxford Inca X-sight energy dispersive spectrometer (EDS) at an accelerating voltage of 200 kV.

The diffuse reflectance UV-vis spectra were obtained using a Shimadzu UV-2501 PC spectrophotometer with an ISR-240A diffuse reflectance unit. Absorption spectra of the methylene blue solutions were recorded on a Cary 100 (Varian) spectrophotometer in the wavelength range 200–800 nm using a quartz cuvette with the optical path length 10 mm; 10 vol% of ethanol in water served as a reference solution.

The chemical state of copper and titanium in the photocatalysts was studied by X-ray photoelectron spectroscopy (XPS). The measurements were performed using an X-ray photoelectron spectrometer (SPECS Surface Nano Analysis GmbH, Germany) equipped with a PHOIBOS-150 hemispherical electron energy analyzer, a XR-50 M X-ray source with a twin Al/Ag anode, and an ellipsoidal crystal monochromator FOCUS-500. The core-level spectra were obtained under ultrahigh vacuum conditions using the monochromatic Al Kα radiation (*hν* = 1486.74 eV). The charge correction was performed by setting the Ti 2p_3/2_ peak at 459.0 eV. In this case, the main peak in the C 1s spectra was observed at 285.2 ± 0.1 eV. Relative concentrations of elements were determined from the integral intensities of the core-level spectra using the cross sections according to Scofield. For detailed analysis, the spectra were fitted into several peaks after the background subtraction by the Shirley method. The fitting procedure was performed using the CasaXPS software. The line shapes of the peaks were approximated by the multiplication of Gaussian and Lorentzian functions.

The XANES measurements were performed at the Structural Materials Science beamline at the Kurchatov Synchrotron Radiation Source (National Research Center “Kurchatov Institute”, Moscow, Russia). The spectra were obtained at the Cu K-edge in the transmission mode using a channel-cut Si(111) monochromator. Two gridded ionization chambers, filled with appropriate N_2_–Ar mixtures, were used as detectors. The ionization currents were measured by Keithley 6487 digital picoamperemeters. The monochromator was calibrated using the first inflection point in the K-edge spectra of samples under study at 8979 eV. The obtained data were analyzed using the ATHENA software.

### 2.3 Photocatalytic test

Catalytic activity of the synthesized photocatalysts was measured in photocatalytic hydrogen production from aqueous-alcoholic solutions of methylene blue. The reactor was loaded with 10 ml of ethanol, 90 ml of an aqueous solution of methylene blue, and 50 mg of the photocatalyst. The reactor was preliminarily purged with argon to achieve a complete oxygen removal and then held in darkness under continuous stirring for 30 min to establish the adsorption–desorption equilibrium.^2,3^ After that, the reactor was irradiated with a 386-LED or 450-LED light emitting diode for 2 h. The concentration of evolved hydrogen was measured by a KHROMOS-1000 gas chromatograph using argon as a carrier gas. The initial concentration of the dye was varied in the experiments from 1.8 to 18 mg L^−1^. Additional experiments were carried out in the absence of ethanol or methylene blue.

## 3 Results and discussion

### 3.1 Characterization of the photocatalysts

**Fig. 1:**
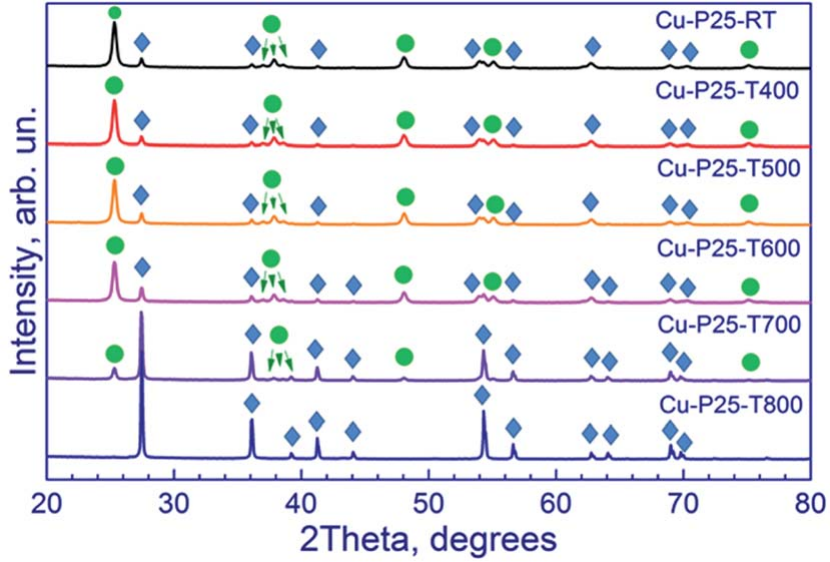
X-ray diffraction patterns of fresh Cu–TiO_2_ photocatalysts. Circle in the figure denotes the anatase peaks (PDF#21-1272), and rhombus denotes the rutile peaks (PDF#21-1276).

X-ray diffraction patterns of the synthesized photocatalysts are shown in Fig. 1. All the samples, except one calcined at 800 °C, are the two-phase mixtures of anatase and rutile. As the calcination temperature of Evonik P25 titania is raised, the rutile reflections increase in intensity, whereas the anatase reflections decrease. Quantitative calculation of the phase composition confirms these data (Table 1). For Cu-P25-RT, Cu-P25-T400, Cu-P25-T500, and Cu-P25-T600, anatase predominates in the samples, whereas in Cu-P25-T700 rutile prevails. The complete conversion of anatase to rutile is reached at 800 °C. The data obtained are consistent with the literature data: earlier it was shown that an intense conversion of anatase to rutile starts at approximately 600 °C (ref. 20) and terminates at 700–800 °C (ref. 12, 20 and 21) depending on the particle size and shape, crystallinity of the samples, the presence of impurities, *etc.* It should be noted that elevation of the calcination temperature is accompanied by an increase in the particle size of titania for both phases due to sintering of the photocatalyst. This is clearly seen for rutile particles: as the calcination temperature of photocatalysts is raised from 600 to 700 °C, crystalline size of rutile increases nearly twofold. This tendency is retained at a further growth of temperature to 800 °C. No reflections of Cu-containing phases were observed in the XRD patterns.

**Table 1:** Composition and physicochemical properties of the synthesized photocatalysts

Sample	Weight content of titania species		CSR of anatase, nm	CSR of rutile, nm	State of Cu, XANES	State of Cu, XPS	Absorption edge, nm	SSA, m^2^ g^−1^	*V*, cm^3^ g^−1^
	Anatase	Rutile							
Cu-P25-RT	85%	15%	19	30	CuO	CuO (35%), Cu_2_O (65%)	388	55	0.48
Cu-P25-T400	85%	15%	19	31	CuO	CuO (55%), Cu_2_O (45%)	417	53	0.43
Cu-P25-T500	82%	18%	20	34	CuO	CuO (45%), Cu_2_O (55%)	419	52	0.49
Cu-P25-T600	75%	25%	21	40	CuO	—	418	55	0.52
Cu-P25-T700	16%	84%	28	74	CuO, Cu_2_O	CuO (35%), Cu_2_O (65%)	427	19	0.07
Cu-P25-T800	0%	100%	—	135	CuO, Cu_2_O	CuO (65%), Cu_2_O (35%)	422	10	0.04

Textural characteristics of the samples were studied using low-temperature adsorption of nitrogen. The quantitative data are listed in Table 1, which shows that the specific surface area and pore volume of the samples (*V*) remain virtually unchanged for Cu-P25-RT, Cu-P25-T400, Cu-P25-T500, and Cu-P25-T600. An increase in the calcination temperature of titania to 700–800 °C leads to a sharp decrease in the specific surface area and pore volume. These data are consistent with the XRD data. At calcination temperatures of 400–600 °C, anatase prevails in the samples; its dimensions are virtually constant in this temperature range, so the textural characteristics remain unchanged. At higher temperatures, rutile prevails in the samples; its sizes sharply increase with temperature elevation, probably due to sintering. Thus, at 700–800 °C the lower specific surface areas and pore volumes of samples are observed. Note that the higher specific surface areas observed for the Cu-P25-T400–Cu-P25-T600 samples may exert a beneficial effect on the photocatalytic activity.

**Fig. 2:**
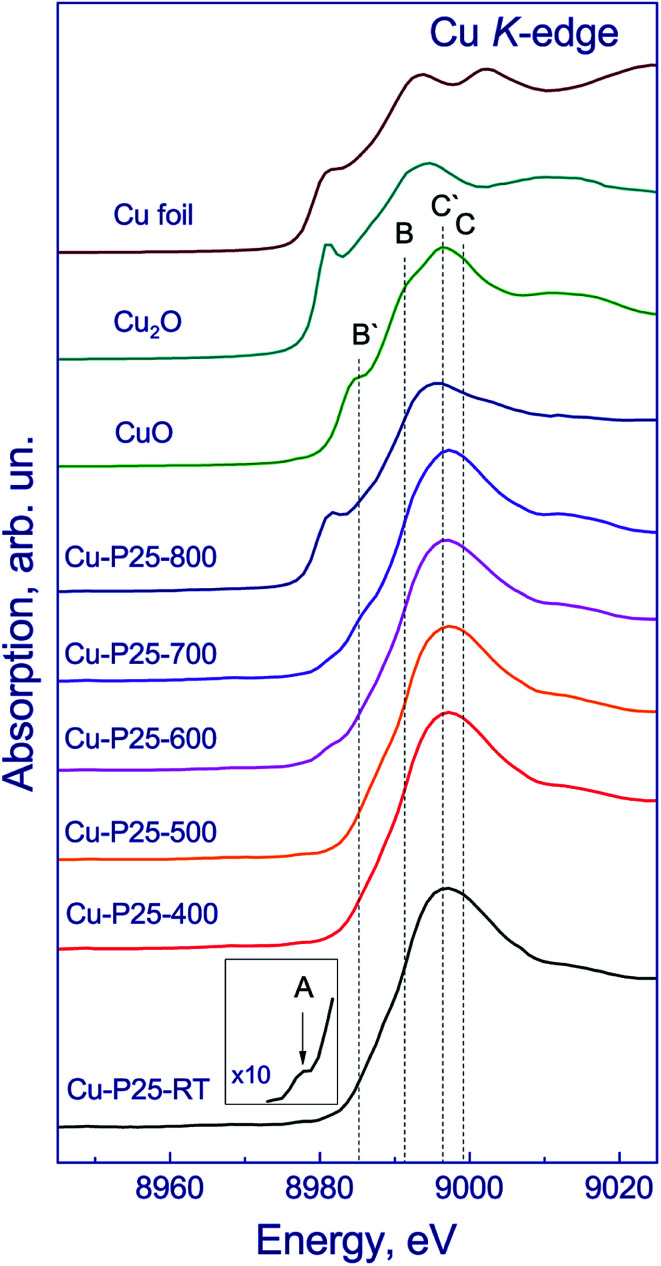
Copper K-edge XANES spectra of the fresh photocatalysts in comparison with the spectra of Cu, Cu_2_O and CuO reference samples.

The chemical state of copper in the fresh photocatalysts was studied by XANES. The copper K-edge XANES spectra of the photocatalysts and bulk copper compounds are shown in Fig. 2. The spectra of the photocatalysts have a broad high-intensity peak at the absorption edge at 8996 eV attributed to the 1s → 4p transitions, which is typical of the spectra of copper in the Cu^2+^ state.^22^ Usually, the spectra of Cu(ii) compounds have a more complex shape. For example, the spectrum of CuO exhibits three different types of peaks between 8970–9000 eV due to the 1s → 3d transition (A), 1s → 4p_*z*_ transition (B), and 1s → 4p_*x*,*y*_ transition (C), which is accompanied by their shakedown satellites B′ and C′.^23–25^ The spectrum of Cu-P25-RT is quite different from that of CuO but similar to that of Cu(OH)_2_ or Cu/ZSM-5.^26,27^ It is known that in a planar or a linear geometry the 1s → 4p_*z*_ transition is not affected by the ligands, therefore, the copper compounds having these geometries exhibit a strong and sharp peak (B) attributed to the 1s → 4p_*z*_ transition, as in CuO.^24,26,27^ The absence of evident peak B in the XANES spectra of Cu-P25-RT indicates that the Cu ions are located in the octahedral symmetry. Similar results were reported by Y. Okamoto *et al.*^28^ for 1%Cu/TiO_2_ photocatalysts. The inset shows the low-intensity pre-peak at 8978 eV in the spectrum of Cu-P25-RT corresponding to the 1s → 3d transition (A). This transition is forbidden by dipole selection rules but shows up due to the 3d–4p orbital mixing. The appearance of such a pre-peak proves that the Cu^2+^ state is present in the sample under consideration because this peak does not appear in the spectra of Cu(i) compounds. Thereby, the copper K-edge XANES spectra of Cu-P25-RT, Cu-P25-T400, Cu-P25-T500, and Cu-P25-T600 are very close to each other, indicating that coper in these photocatalyst is in the Cu^2+^ state and copper cations are in the octahedral coordination environment mainly.

In addition, in the spectrum of Cu-P25-T700, a shoulder at 8986 eV appears, and in the spectrum of Cu-P25-T800 the flat absorption edge turns into a stepwise edge with a distinct shoulder at 8981 eV, and the peak at the absorption edge shifts to 8988 eV, which is typical of copper in the Cu^1+^ state.^22^ The spectra of Cu-P25-T700 and Cu-P25-T800 were simulated by a linear combination of the spectra of Cu foil, Cu_2_O, CuO, and CuO/TiO_2_. The spectrum of Cu-P25-RT was taken as the spectrum of Cu^2+^/TiO_2_ reference sample. According to fitting of the copper K-edge XANES spectrum of Cu-P25-T700, approximately 10% of copper is in the Cu^1+^ state and 90% is represented by Cu^2+^; in the Cu-P25-T800 photocatalyst copper is in the Cu^1+^ state increases to 65% and approximately 35% remains in the Cu^2+^ state.

Hence, the study of the samples with deposited copper allows a conclusion that Cu-P25-T400, Cu-P25-T500, and Cu-P25-T600 contain copper in the Cu^2+^ state mainly, while in the Cu-P25-T800 and Cu-P25-T700 samples copper is certainly present as Cu^1+^ and Cu^2+^. Note that the presence of copper in the oxide state instead of the expected metallic state may be related to the oxidation of copper nanoparticles with air.^29^ Earlier, it was shown that in case of anatase, copper was predominantly oxidized to CuO.^30^ In this work we also observed CuO in the samples with high content of anatase (Cu-P25-T400, Cu-P25-T500, and Cu-P25-T600). Data on the chemical state of copper are listed in Table 1.

**Fig. 3:**
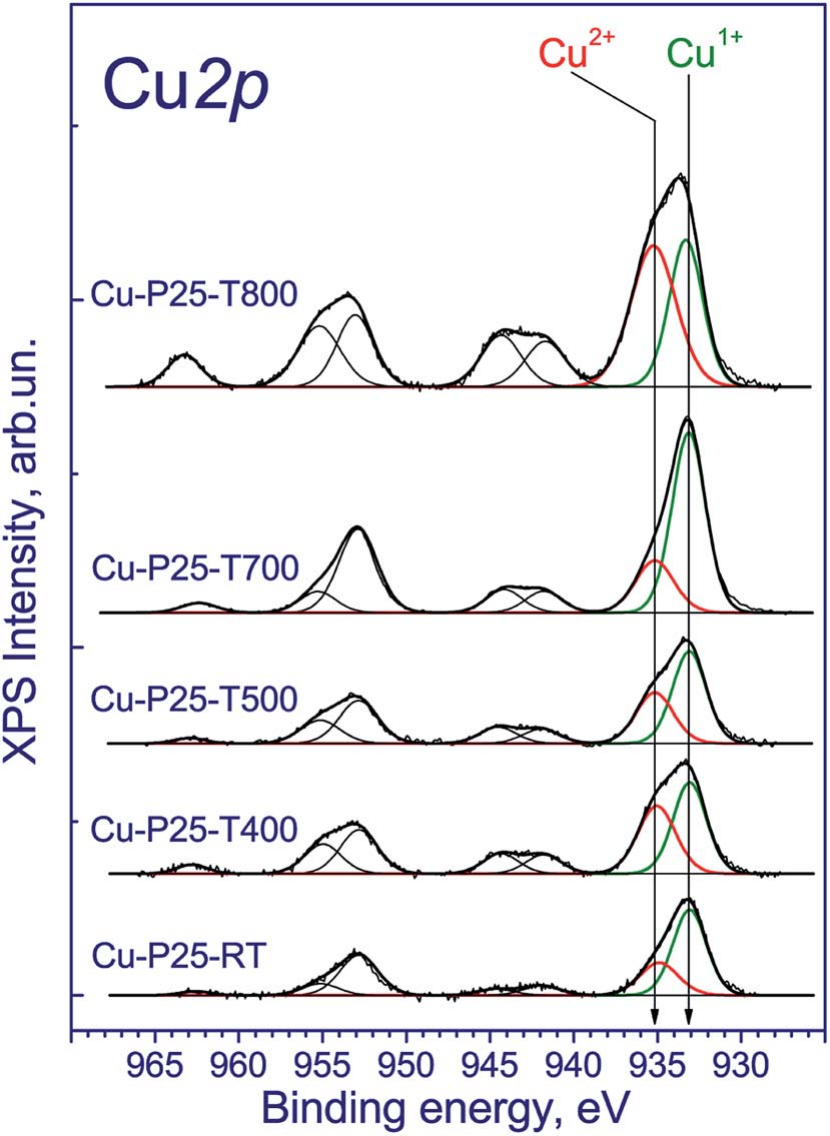
Cu 2p core-level spectrum of fresh Cu–TiO_2_ photocatalysts.

The copper-contained photocatalysts additionally were studied by XPS. The Ti 2p_3/2_ binding energy was 459.0 eV, which corresponds to the Ti^4+^ state;^31^ the O 1s binding energy was 530.3 eV, which confirms the presence of the lattice oxygen species O^2−^. Of particular interest is the chemical state of copper. The Cu 2p spectrum is described by two spin–orbital doublets with the Cu 2p_3/2_ binding energies at 933.1 and 935.1 eV and the corresponding peaks of shake-up satellites at 943.0 and 963.3 eV (Fig. 3). The presence of such intense satellites, which are determined by multielectron processes, is typical of oxides and hydroxides of divalent copper.^32,33^ At the same time, these satellites are absent in the spectra of metallic copper and Cu^1+^ compounds. According to the literature, the Cu 2p_3/2_ binding energy for metallic copper is in the range of 932.5–932.7 eV; for copper in the Cu^1+^ state (Cu_2_O) is in the range of 932.4–932.5 eV; and for Cu^2+^ copper in the CuO structure is in the range of 933.6–934.2 eV.^34–36^ In our case, the Cu 2p_3/2_ peak at 932.7 eV is observed (Fig. 3) and can be attributed to Cu^1+^ and/or Cu^0^ states.^34–36^ Additionally, the Cu 2p spectra have two intense Cu 2p_3/2_ and Cu 2p_1/2_ peaks at 933.1–933.2 and 953.1–953.2 eV without shake-up satellites. Because of the similar binding energies of Cu^1+^ and Cu^0^ states, the Auger-parameter *α*, which is equal to the sum of the Cu 2p_3/2_ binding energy and the Cu-LMM peak kinetic energy,^37^ was used for the identification of the copper states. According to the literature,^38–42^ the Auger-parameters of metallic Cu, Cu_2_O, and CuO are in the ranges of 1851.0–1851.4, 1848.7–1849.3, and 1851.4–1851.7 eV, respectively. The Auger-parameter of the catalysts for Cu^2+^ species is 1851.8 eV, which is close to one for CuO. For the second species the Auger-parameter is 1848.7 eV that corresponds to Cu^1+^ species. Hence, we can conclude that copper in the fresh catalysts is mainly in the Cu^2+^ and Cu^1+^ state. It should be noted that there are some differences between the XANES and XPS results. The increased content of Cu^1+^ may be associated with the partial reduction of Cu^2+^ to Cu^1+^ under the influence of soft X-ray radiation during the recording of X-ray photoelectron spectra, which is typical for highly dispersed CuO species.

**Fig. 4:**
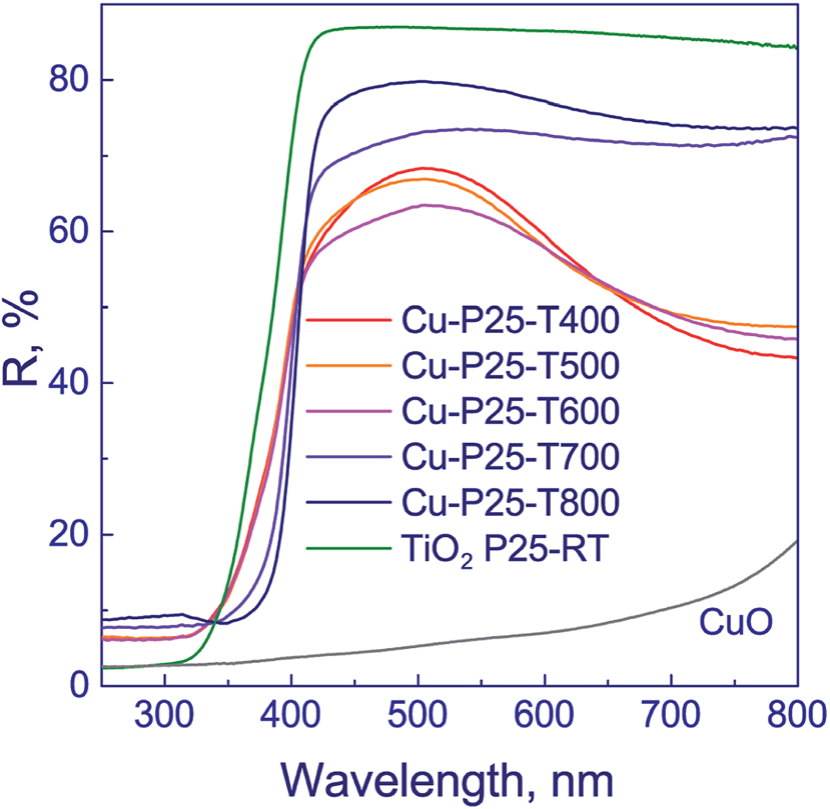
Diffuse reflectance spectra of fresh Cu–TiO_2_ photocatalysts.

Optical properties of the photocatalysts were examined by diffuse reflectance spectroscopy (Fig. 4). The absorption edge of anatase is known to be *ca.* 390 nm,^37^ and that of rutile, 410 nm.^43,44^ The deposition of copper on titania surface shifts the absorption edges of photocatalysts to the visible range to 427 nm (Table 1). This may occur due to the electron density transfer from the TiO_2_ conduction band to the particles of co-catalyst. It should be noted that, as shown in Fig. 4, the profiles of reflectance spectra for photocatalysts that were synthesized using titania calcined at 400–600 °C are virtually identical. The reflectance bands observed in the region of 320–400 nm are typical of titania. The reflectance spectra of photocatalysts that were synthesized using titania calcined at 700–800 °C have similar profiles. At 350–400 nm wavelengths, the reflectance band of titania is observed. Therewith, for the series of samples calcined at 400–600 and 700–800 °C, the bands corresponding to intense reflectance of titania are shifted relative to each other, as shown in Fig. 4. The observed effect may be attributed to the prevalence of anatase in the Cu-P25-T400, Cu-P25-T500, and Cu-P25-T600 photocatalysts, and rutile in the Cu-P25-T700 and Cu-P25-T800 photocatalysts, which are characterized by different values of absorption edges. A decrease in reflectivity in the region of 500–800 nm for all samples is related to the presence of copper(ii) oxide in the sample.^45,46^ Nevertheless, in the case of titania calcination at 400–600 °C, the presence of CuO in the reflectance spectra is more distinct, probably owing to a higher content of Cu^2+^. Thus, data obtained by diffuse reflectance spectroscopy are consistent with the XRD and XANES data.

**Fig. 5:**
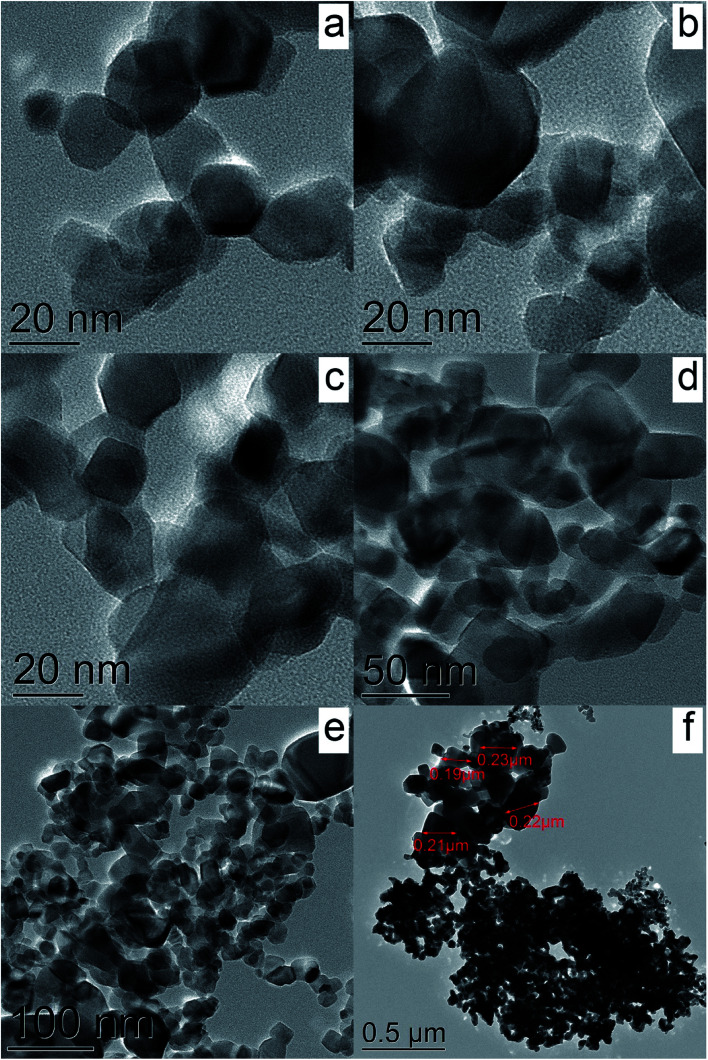
TEM images of the samples Cu-P25-RT (a), Cu-P25-T400 (b), Cu-P25-T500 (c), and Cu-P25-T800 (d–f).

Fig. 5a–f show TEM images of the Cu-P25-RT, Cu-P25-T400, Cu-P25-T500, and Cu-P25-T800 photocatalysts. One can see, that, as expected, the TEM images of the first three samples are very similar to each other, well crystallized titanium dioxide nanoparticles with a size of 20–40 nm can be identified. The Cu-P25-T800 photocatalyst consists of the particle with a higher size; some particles are approximately 200 nm in size. Surprisingly, no Cu-containing nanoparticles were found by TEM in all the samples. However, EDS analysis undoubtedly proves the presence of copper which homogeneously distributed over titanium dioxide; the amount of copper corresponded to approximately 1 wt%. Taking into account the XRD data, we can conclude that copper oxides (CuO_*x*_, *x* = 0.5–1) exist over the titania surface as clusters with a size less than 1 nm.

### 3.2 Photocatalytic hydrogen production

All the synthesized photocatalysts were studied in the photocatalytic hydrogen production from aqueous solutions of methylene blue with different content of the dye. Gas chromatography did not reveal hydrogen production when the system was exposed to irradiation with the 386 and 450 nm wavelengths. Then aqueous solution of the dye was supplemented with ethanol as a sacrificial reagent, and the reaction rate was measured. When visible light was used, the reaction rate was again equal to zero, whereas UV radiation made it possible to evolve hydrogen in the tested system. Thus, in our case, methylene blue did not produce a sensitizing effect on titania.

**Fig. 6:**
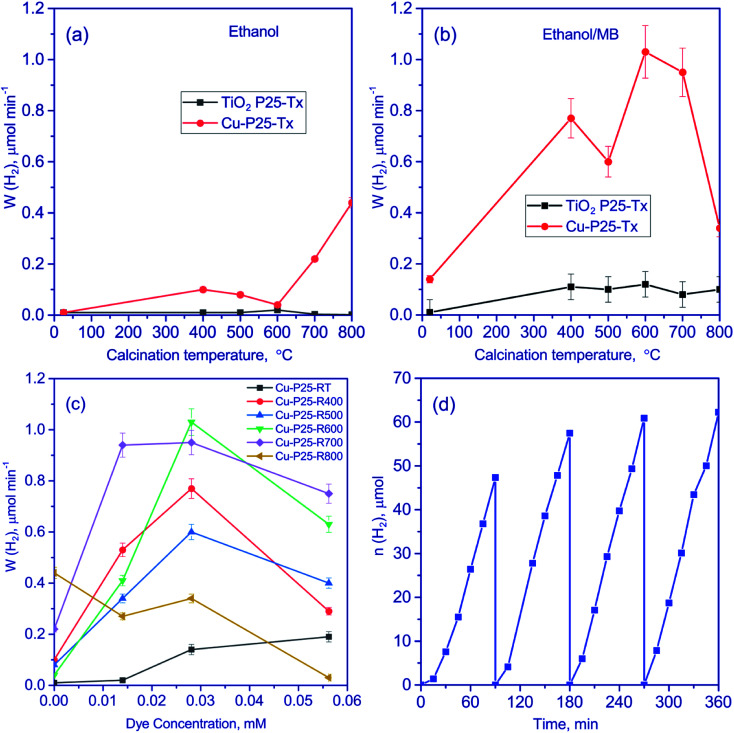
The effect of calcination temperature for the samples P25-T*x* and Cu-P25-T*x* (*T* = 400–800) from ethanol (a) and aqueous-alcoholic solutions of methylene blue (*C*_0_ (MB) = 2.81 × 10^−5^ M) (b) solution; the effect of methylene blue concentration on the rate of photocatalytic hydrogen production from aqueous-alcoholic solutions of methylene blue (c); cyclic experiments on hydrogen evolution over Cu-P25-T400 from aqueous-alcoholic solutions of methylene blue (*C*_0_ (MB) = 2.81 × 10^−5^ M) (d). Conditions of experiments: 10 vol% of ethanol, 50 mg of photocatalyst, volume of suspension 100 ml, the process temperature 20 °C, 386-LED as a source of light.

Quantitative data of the experiments performed under UV irradiation are listed in Fig. 6. It is seen that the presence of ethanol makes it possible to increase the rate of photocatalytic hydrogen production, probably owing to a greater involvement of electrons and holes in the chemical transformations (Fig. 6a). A similar approach was earlier applied to the photocatalytic hydrogen production from water.^1^ It can be seen that the deposition of copper significantly increases the rate of hydrogen production. This may be related to the presence of Cu_2_O and CuO in the photocatalysts and the appearance of heterojunctions between oxides of univalent and divalent copper and titania nanoparticles. According to the literature,^45–47^ the presence of such heterojunctions improves the spatial separation of charge carriers, thus increasing the lifetime of photogenerated electrons and enhancing the catalytic activity. Recently, M. G. Méndez-Medrano and co-workers^48^ have proved the formation of heterojunctions in CuO–TiO_2_ systems. Note, that according to the TEM data, the particle size of copper oxide species is very low, thus the formation of heterojunctions between Cu_2_O, CuO, and TiO_2_ seems to be efficient.

The rate of the hydrogen production over Cu-P25-RT is very low. All samples which were synthesized with the thermal treatment stage possessed higher activity in ethanol aqueous solutions. Recently, it has been shown,^19^ that the thermal activation of titania in the temperature range between 300 and 600 °C leads to an increase in photoreactivity of platinized titania in the hydrogen production. According to the XPS study, this effect is due to formation of cationic vacancies that limit the fast electron–hole recombination.^19^ Also, increased content of rutile can improve the activity of such complicated Cu_2_O/CuO/TiO_2_ photocatalysts.

Then we analyzed the data on the hydrogen production from aqueous solutions containing both ethanol and methylene blue (Fig. 6b and c). With the addition of methylene blue, the rate of the hydrogen production grows for all the photocatalysts except of Cu-P25-T800 (Fig. 6c). In the case of the Cu-P25-T700 and Cu-P25-T800 photocatalysts having a high content of rutile, the hydrogen production rate goes to a plateau in the concentration range of 1.40 × 10^−5^ to 2.81 × 10^−5^ M. In this region, the reaction rate changes by less than 5%. For the samples with a high content of anatase, Cu-P25-RT–Cu-P25-T600, a considerable increase in the reaction rate was observed with the increase of the dye concentration from 0 to 2.81 × 10^−5^ M. The observed effect may be related to different adsorptivity of the polytropic modifications of titania. It should be noted that the specific surface area of the Cu-P25-T700 and Cu-P25-T800 photocatalysts is lower by a factor of 3–5 as compared to the Cu-P25-T400–Cu-P25-T600 samples; this may affect also the adsorptivity and photocatalytic activity of the samples. A further growth of the dye concentration decreased the hydrogen production rate for all samples due to optical effects. The pristine titania calcinated at different temperature also was studied in the target reaction at an optimal dye concentration (Fig. 6b). The reaction rate was quite low for all calcination temperatures comparing Cu-contained photocatalysts. The reason of the low activity is described above.

The highest reaction rates were obtained for the Cu-P25-T600 and Cu-P25-T700 photocatalysts. The highest catalytic activity was observed for Cu-P25-T600 (the concentration of methylene blue 2.81 × 10^−5^ M); it was equal to 1.24 mmol (h^−1^ g^−1^). Thus, using aqueous solutions containing both dye and ethanol, it is possible to achieve much higher rates than using individual solutions. To the best of our knowledge, that similar experiments on hydrogen production from aqueous-alcoholic solution of methylene blue were described in only sole work.^17^ However, the reaction rate of these processes was extremely low, 0.03 mmol (h^−1^ g^−1^) only.

For photocatalytic applications, long-time stability is very important as well as the photocatalytic activity. We tested the activity of the Cu-P25-T400 photocatalyst in aqueous solutions containing both dye and ethanol in four 1.5 h consecutive photocatalytic runs (Fig. 6d). One can see that the rate increases in the first cycle and then remains almost constant, which is a very promising result. The activation of the sample would be further explained by means of XPS experiments.

**Fig. 7:**
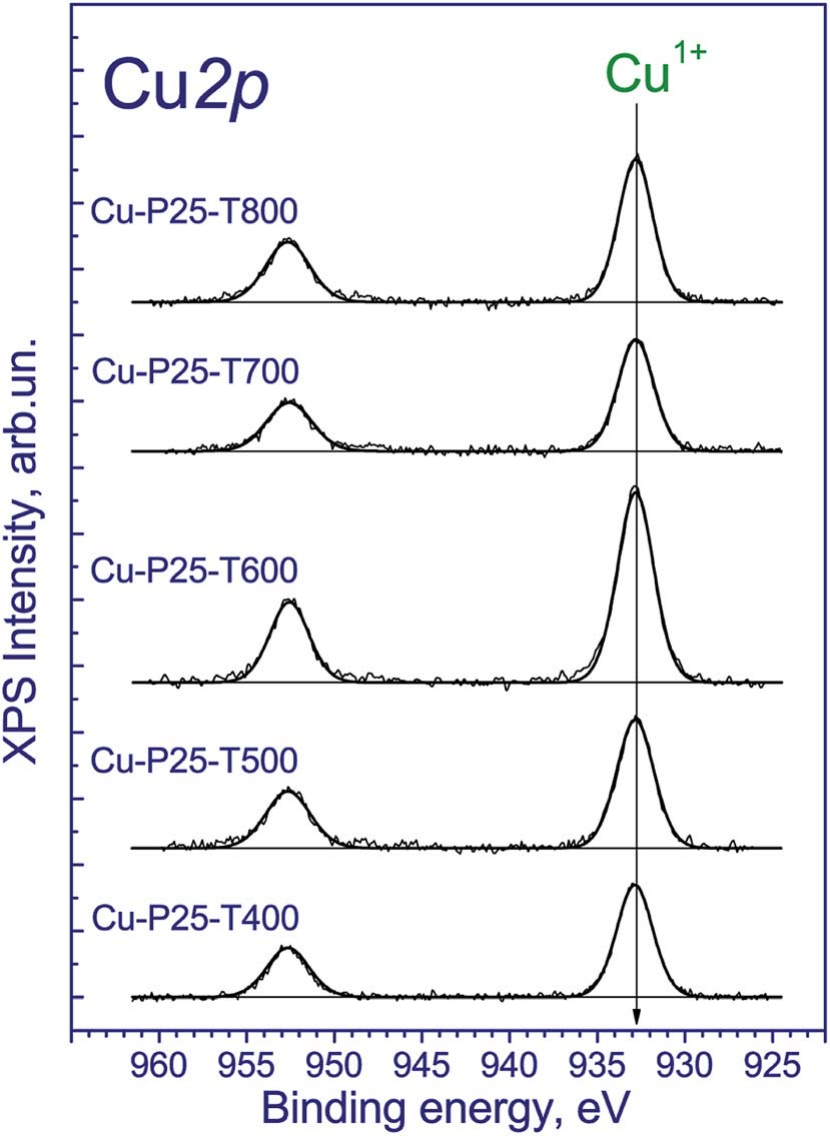
Cu 2p core-level spectra of the tested photocatalysts.

To understand the nature of processes that occur during photocatalytic production of hydrogen, the photocatalysts after irradiation of the aqueous-alcoholic solution with the highest concentration of the dye were examined by XPS. The Ti 2p_3/2_ and O 1s binding energies have the same values as all the samples before catalytic testing. Of particular interest is the chemical state of copper on the photocatalyst surface (Fig. 7). The obtained spectra have two narrow peaks at 932.7 and 952.7 eV corresponding to Cu 2p_3/2_ and Cu 2p_1/2_ core levels. The Cu 2p_3/2_ peak at 932.7 eV can be attributed to the Cu^1+^ and/or Cu^0^ states.^34–36^ Indeed, because the Cu 2p_3/2_ binding energies of the Cu^1+^ and Cu^0^ states are similar, it hampers their identification. Nevertheless, the Auger-parameter α, which is equal to the sum of the Cu 2p_3/2_ peak binding energy and the Cu LMM peak kinetic energy,^37^ can be used for the identification of Cu^1+^ and Cu^0^ states. According to the literature,^40–42^ the Auger parameters of metallic Cu, Cu_2_O, and CuO are in the ranges of 1851.0–1851.4, 1848.7–1849.3, and 1851.4–1851.7 eV, respectively. The Auger parameter of the catalysts is 1848.0 eV, which is close to one of Cu^1+^. Thus, along with the photocatalytic hydrogen production, the reduction of divalent copper to univalent proceeds on the photocatalyst surface. Note that the same effect was earlier observed for Cu^2+^ salts adsorbed on the titania surface and illuminated under UV-light in argon atmosphere.^49^ The reduction of copper is likely the reason of the activation of the Cu-P25-T400 photocatalyst in the cyclic experiments. Note that after long-term experiment with the sample Cu-P25-T400, the copper in the final Cu 2p spectrum was in the Cu^1+^ state only.

**Fig. 8:**
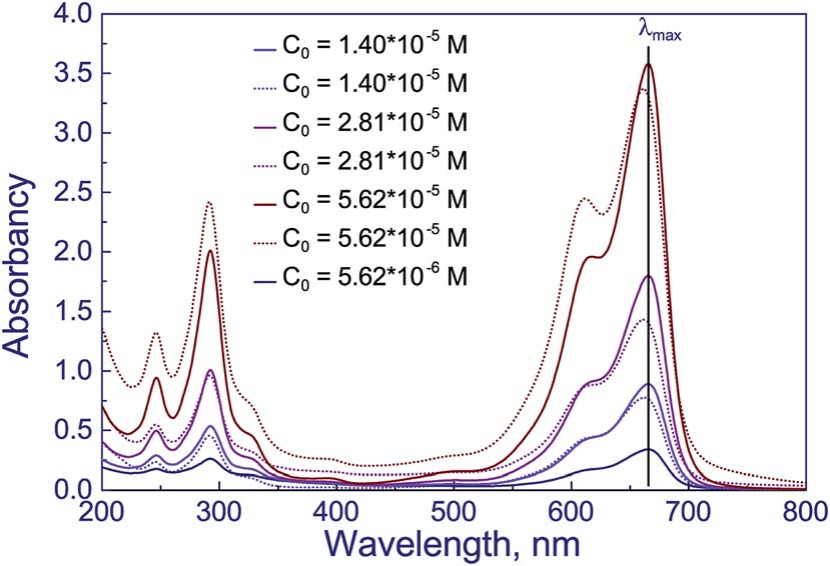
UV-vis spectra of water–ethanol solutions of methylene blue before (solid lines) and after photocatalytic reaction over Cu/T400 (dot lines).

To elucidate the processes accompanying the photocatalytic reaction with the dye solution, methylene blue solutions were studied by optical spectroscopy (Fig. 8). In Fig. 8, solid lines correspond to the spectra of the dye solution before the reaction. One can see three peaks in these spectra: at 246, 292, and 666 nm. Peaks in the region of 240–300 nm, according to the literature data, are assigned to polycyclic aromatic compounds,^50^ while the peak at 666 nm corresponds to n–π* transitions in the molecule of methylene blue monomer.^50,51^ The shoulder near 612 nm corresponds to the absorption of dimeric form of the dye.^50^ The highest optical density is observed at a wavelength of 666 nm; this value was used for quantitative measurements.

**Fig. 9:**
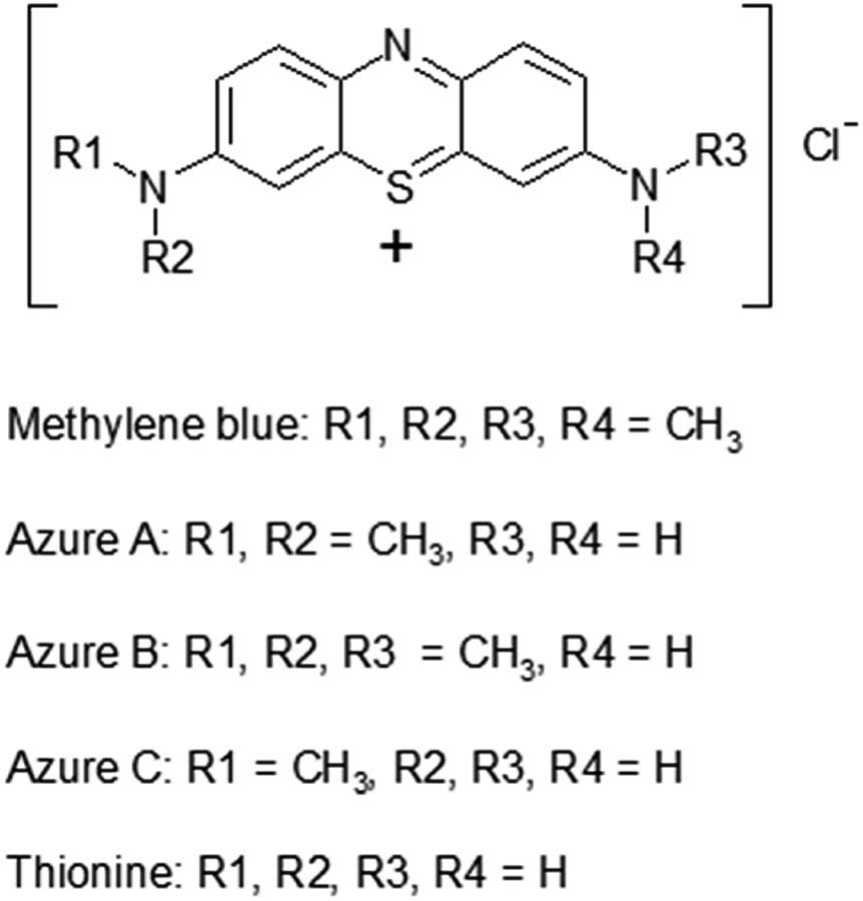
Structures of some thiazine dyes.

In Fig. 8, the optical spectra of methylene blue dye after photocatalytic reaction on the Cu-P25-T400 sample are indicated by dotted lines. It is seen that the absorption maximum is shifted to the blue spectral region approximately by 5 nm. This may be caused by the formation of new chemical compounds from the dye molecule. According to the literature, high performance liquid chromatography revealed the formation of some dyes of the thiazine series, such as azures A, B, C and thionine (their structural formulas are displayed in Fig. 9), during irradiation of methylene blue solutions in the presence of modified titania^52^ and manganese oxide.^53^ For all these dyes, the absorption peak in visible region is shifted, with azure B having the least difference between positions of the absorption maxima of methylene blue and other dyes.^54^ Azure B was earlier identified by optical spectroscopy as a product of oxidative demethylation of methylene blue in the presence of double layer hydroxides.^55^ Thus, the first step in photocatalytic conversion of the dye in the presence of various photocatalysts is oxidative demethylation.

Optical densities of the dye solutions before and after irradiation at a wavelength of 666 nm were used to calculate the decoloration degree of the dye. For solutions with the initial concentrations of methylene blue 1.40 × 10^−5^, 2.82 × 10^−5^, and 5.63 × 10^−5^ M, in the presence of Cu-P25-T400 this value was 14, 22, and 8%, respectively. It should be noted that changes in decoloration degree and hydrogen production rate with increasing the initial dye concentration follow a similar trend. Moreover, the quantitative proportions are also retained: as the dye concentration is raised from 1.40 × 10^−5^ to 2.82 × 10^−5^ M, decoloration degree of methylene blue solutions increases 1.6-fold, and hydrogen production rate, 1.5-fold. A further growth of the dye concentration to 5.63 × 10^−5^ M decreases the decoloration degree of methylene blue by a factor of 2.8 and the rate of photocatalytic hydrogen production by a factor of 2.7. Thus, a correlation exists between photocatalytic hydrogen production rate and decoloration degree of the dye solution; this may be caused by the formation of hydrogen as the dye demethylation byproduct.

A comparison of hydrogen production rates at different concentrations of the dye and in its absence allows a conclusion that the reaction rate is higher on virtually all the photocatalysts in case of the simultaneous presence of methylene blue and ethanol. For example, for the Cu-P25-T600 photocatalyst, the rate of reaction from the aqueous solution of dye is equal to zero; from the alcohol solution, 0.04 μmol min^−1^; whereas at the optimal concentration of methylene blue in dye/ethanol solution this value increases 25-fold. Note that the introduction of the sacrificial reagent in the system often enhances the photocatalytic activity. Several types of processes involving photogenerated electrons and holes occur on the surface of photocatalysts. Electrons reduce water to hydrogen and hydroxide ions, while holes enter the oxidation reactions. The introduction of ethanol as a sacrificial reagent in the system changes the mechanism of photocatalytic process.^56^ Photogenerated holes can participate in the oxidation of alcohol,^57^ thus increasing the degree of spatial separation of charge carriers. In addition, ethanol can serve as a source for the hydrogen production.^57^ Moreover, the experiments with deuterated water and methanol revealed that during the photocatalytic hydrogen production from aqueous solution of monatomic alcohols hydrogen is evolved from both the water and the alcohol.^57^ The addition of methylene blue to the aqueous solution of ethanol may produce similar effects. The appearance of another substrate leads to the oxidation of ethanol and the dye molecule in the system, thus increasing the degree of spatial separation of the electron–hole pairs. Irradiation initiates the oxidative demethylation of methylene blue, which leads to hydrogen production. This is indirectly indicated by quantitative changes in the decoloration degree of methylene blue solutions and the rate of photocatalytic hydrogen production upon variation of the initial concentration of the dye. Spatial separation of charge carriers and efficiency of photocatalytic processes are facilitated also by the transformation of the CuO co-catalyst to Cu_2_O, which involves photoinduced electrons. Thus, the simultaneous presence of two sacrificial reagents – ethanol and methylene blue – is one of the conditions for efficient photocatalytic hydrogen production.

## 4 Conclusions

In this work, photocatalysts containing titania and copper oxides (CuO_*x*_/TiO_2_) were studied. The synthesis of the photocatalysts included the thermal treatment of commercial TiO_2_ P25 in the range between 400 to 800 °C. All photocatalysts consist of titania nanoparticles and highly dispersed species of copper oxides; this structure contributes to the formation of heterojunctions which improves the spatial separation of charge carriers, thus increasing the lifetime of photogenerated electrons and enhancing the catalytic activity. It was shown that the thermal treatment affects both phase composition of titanium dioxide and copper oxidation state.

Photocatalytic activity of the samples was investigated in the hydrogen production from aqueous and aqueous-alcoholic solutions of methylene blue under the action of UV radiation (*λ* = 386 nm). Hydrogen production was not observed in the absence of alcohol whereas for ethanol solution. Efficient hydrogen evolution was observed in the case of the aqueous-alcoholic solutions of the dye; the reaction rate increased with raising the dye concentration up to 2.8 × 10^−5^ M. A further growth of the dye concentration decreased the hydrogen production rate due to optical effects. The highest photoreactivity was observed for the Cu-P25-T600 sample (the concentration of methylene blue 2.81 × 10^−5^ M) and reached 1.24 mmol (h^−1^ g^−1^). The high activity of the photocatalyst calcined at 600 °C is explained by a combination of factors, such as the phase and defect structure of titanium dioxide and the oxidation state of copper. The simultaneous presence of ethanol and methylene blue was shown to provide the most efficient production of hydrogen and the removal of methylene blue impurities from solutions.

## Funding

This work was supported by Russian Science Foundation (grant #19-73-20020).

## Conflicts of interest

Authors declare they do not have any conflict of interest.

## Supplementary Material

## References

[cit1] Kozlova E. A., Parmon V. N. (2017). Heterogeneous semiconductor photocatalysts for hydrogen production from aqueous solutions of electron donors. Russ. Chem. Rev..

[cit2] Kangralkar M. V., Kangralkar V. A., Momin N., Manjanna J. (2019). Cu_2_O nanoparticles for removal of methylene blue dye from solution. Environmental Nanotechnology, Monitoring and Management.

[cit3] Liu H., Zhong L., Govindaraju S., Yun K. (2019). ZnO rod decorated with Ag nanoparticles for enhanced photocatalytic degradation of methylene blue. J. Phys. Chem. Solids.

[cit4] Sawafta R., Shahwan T. (2019). A comparative study of the removal of methylene blue by iron nanoparticles from water and water–ethanol solutions. J. Mol. Liq..

[cit5] Tcher S., Sullivan J. B. (2020). Clinical utility of midodrine and methylene blue as catecholamine-sparing agents in intensive care unit patients with shock. J. Crit. Care.

[cit6] Pourreza N., Abdollahzadeh R. (2019). Colorimetric determination of hydrazine and nitrite using catalytic effect of palladium nanoparticles on the reduction reaction of methylene blue. Microchem. J..

[cit7] Clark W. M., Cohen B., Gibbs H. D. (1925). Studies on Oxidation-Reduction: VIII. Methylene Blue. Public Health Rep..

[cit8] Moreno-Castilla C. (2004). Adsorption of organic molecules from aqueous solutions on carbon materials. Carbon.

[cit9] Pavan F. A., Mazzocato A. C., Gushikem Y. (2008). Removal of methylene blue dye from aqueous solution by adsorption using yellow passion fruit peel as adsorbent. Bioresour. Technol..

[cit10] Beluci N. D. L., Mateus G. A. P., Miyashiro C. S., Homem N. C., Gomes R. G., Fagundes-Klen M. R., Bergamasco R., Vieira A. M. S. (2019). Hybrid treatment of coagulation/flocculation process followed by ultrafiltration in TiO_2_-modified membranes to improve the removal of reactive black 5 dye. Sci. Total Environ..

[cit11] Nidheesh P. V., Zhou M., Oturan M. A. (2018). An overview on the removal of synthetic dyes from water by electrochemical advanced oxidation processes. Chemosphere.

[cit12] Carp O., Huisman C. L., Reller A. (2004). Photoinduced reactivity of titanium dioxide. Prog. Solid State Chem..

[cit13] Syoufian A., Nakashima K. (2008). Degradation of methylene blue in aqueous dispersion of hollow titania photocatalyst: study of reaction enhancement by various electron scavengers. J. Colloid Interface Sci..

[cit14] Suo G., Li D., Feng L., Hou X., Ye X., Zhang L., Yu Q., Yang Y., Wang W. (2020). Construction of SnS_2_/SnO_2_ heterostructures with enhanced potassium storage performance. J. Mater. Sci. Technol..

[cit15] Li D., Zhang J., Ahmed S. M., Suo G., Wang W., Feng L., Hou X., Yang Y., Ye X., Zhang L. (2020). Amorphous carbon coated SnO_2_ nanohseets on hard carbon hollow spheres to boost potassium storage with high surface capacitive contributions. J. Colloid Interface Sci..

[cit16] Suo G., Zhang J., Li D., Yu Q., He M., Feng L., Hou X., Yang Y., Ye X., Zhang L., Wang W. (2020). Flexible N doped carbon/bubble-like MoS_2_ core/sheath framework: buffering volume expansion for potassium ion batteries. J. Colloid Interface Sci..

[cit17] Simamora A.-J., Hsiung T.-L., Chang F.-C., Yang T.-C., Liao C.-Y., Wang H. P. (2012). Photocatalytic splitting of seawater and degradation of methylene blue on CuO/nano TiO_2_. Int. J. Hydrogen Energy.

[cit18] Su R., Bechstein R., Sø L., Vang R. T., Sillassen M., Esbjörnsson B., Palmqvist A., Besenbacher F. (2011). How the anatase-to-rutile ratio influences the photoreactivity of TiO_2_. J. Phys. Chem. C.

[cit19] Kurenkova A. Y., Kremneva A. M., Saraev A. A., Murzin V., Kozlova E. A., Kaichev V. V. (2020). Influence of Thermal Activation of Titania on Photoreactivity of Pt/TiO_2_ in Hydrogen Production. Catal. Lett..

[cit20] Hanaor D. A. H., Sorrell C. C. (2011). Review of the anatase to rutile transformation. J. Mater. Sci..

[cit21] Byrne C., Fagan R., Hinder S., McCormack D. E., Pillai S. C. (2016). New approach of modifying the anatase to rutile transition temperature in TiO_2_ photocatalysts. RSC Adv..

[cit22] Hsiung T. L., Wang H. P., Lu Y.-M., Hsiao M. C. (2006). *In situ* XANES studies of CuO/TiO_2_ thin films during photocatalytic degradation of CHCl_3_. Radiat. Phys. Chem..

[cit23] Kosugi N., Yokoyama T., Asakura K., Kuroda H. (1984). Polarized Cu K-edge XANES of square planar CuCl_4_^2−^ ion. Experimental and theoretical evidence for shake-down phenomena. Chem. Phys..

[cit24] Kau L. S., Spira-Solomon D. J., Penner-Hahn J. E., Hodgson K. O., Solomon E. I. (1987). X-ray absorption edge determination of the oxidation state and coordination number of copper. Application to the type 3 site in Rhus vernicifera laccase and its reaction with oxygen. J. Am. Chem. Soc..

[cit25] Kau L. S., Hodgson K. O., Solomon E. I. (1989). X-ray absorption edge and EXAFS study of the copper sites in zinc oxide methanol synthesis catalysts. J. Am. Chem. Soc..

[cit26] Aritani H., Akasaka N., Tanaka T., Funabiki T., Yoshida S., Gotoh H., Okamoto Y. (1996). Reduction of NO over TiO_2_-supported Cu catalysts. J. Chem. Soc., Faraday Trans..

[cit27] Groothaert M. H., van Bokhoven J. A., Battiston A. A., Weckhuysen B. M., Schoonheydt R. A. (2003). Bis(μ-oxo)dicopper in Cu-ZSM-5 and its role in the decomposition of NO: a combined *in situ* XAFS, UV-Vis-Near-IR, and kinetic study. J. Am. Chem. Soc..

[cit28] Okamoto Y., Gotoh H., Hishida K., Aritani H., Tanaka T., Yoshida S. (1997). Surface copper-TiO_2_ interaction species for NO·CO reactions. Appl. Surf. Sci..

[cit29] Mendez-Medrano M. G., Kowalska E., Lehoux A., Herissan A., Ohtani B., Bahena D., Briois V., Colbeau-Justin C., Rodriguez-Lopez J. L., Remita H. (2016). Surface modification of TiO_2_ with Ag nanoparticles and CuO nanoclusters for application in photocatalysis. J. Phys. Chem. C.

[cit30] Ajmal A., Majeed I., Malik R. N., Iqbal M., Nadeem M. A., Hussain I., Yousuf S. Z., Mustafa G., Zafar M. I., Nadeem M. A. (2016). Photocatalytic degradation of textile dyes on Cu_2_O–CuO/TiO_2_ anatase powders. J. Environ. Chem. Eng..

[cit31] Finetti P., Sedona F., Rizzi G. A., Mick U., Sutara F., Svec M., Matolin V., Schierbaum K., Granozzi G. (2007). Core and valence band photoemission spectroscopy of well-ordered ultrathin TiO_*x*_ films on Pt (111). J. Phys. Chem. C.

[cit32] Svintsitskiy D. A., Stadnichenko A. I., Demidov D. V., Koscheev S. V., Boronin A. I. (2011). Investigation of oxygen
states and reactivities on a nanostructured cupric oxide surface. Appl. Surf. Sci..

[cit33] Biesinger M. C., Lau L. W. M., Gerson A. R., Smart R. S. C. (2010). Resolving surface chemical states in XPS analysis of first row transition metals, oxides and hydroxides: Sc, Ti, V, Cu and Zn. Appl. Surf. Sci..

[cit34] Skinner W. M., Prestidge C. A., Smart R. S. C. (1996). Irradiation effects during XPS studies of Cu(ii) activation of zinc sulphide. Surf. Interface Anal..

[cit35] McIntyre N. S., Sunder S., Shoesmith D. W., Stanchell F. W. (1981). Chemical information from XPS-applications to the analysis of electrode surfaces. J. Vac. Sci. Technol..

[cit36] Chawla S. K., Sankarraman N., Payer J. H. (1992). Diagnostic spectra for XPS analysis of Cu–O–S–H compounds. J. Electron Spectrosc. Relat. Phenom..

[cit37] Moretti G. (1995). Auger parameter and wagner plot in the characterization of chemical states: initial and final state effects. J. Electron Spectrosc. Relat. Phenom..

[cit38] Bukhtiyarov V. I., Kaichev V. V., Prosvirin I. P. (2005). X-ray photoelectron spectroscopy as a tool for *in situ* study of the mechanisms of heterogeneous catalytic reactions. Top. Catal..

[cit39] Strohmeier B. R., Levden D. E., Field R. S., Hercules D. M. (1985). Surface spectroscopic characterization of CuAl_2_O_3_ catalysts. J. Catal..

[cit40] Poulston S., Parlett P. M., Stone P., Bowker M. (1996). Surface oxidation and reduction of CuO and Cu_2_O studied using XPS and XAES. Surf. Interface Anal..

[cit41] Batista J., Pintar A., Mandrino D., Jenko M., Martin V. (2011). XPS and TPR examinations of γ-alumina-supported Pd–Cu catalysts. Appl. Catal., B.

[cit42] Richter M., Fait M., Eckelt R., Schneider M., Radnik J., Heidemann D., Fricke R. (2007). Gas-phase carbonylation of methanol to dimethyl carbonate on chloride-free Cu-precipitated zeolite Y at normal pressure. J. Catal..

[cit43] Ding L., Yang S., Liang Z., Qian X., Chen X., Cui H., Tian J. (2020). TiO_2_ nanobelts with anatase/rutile heterophase junctions for highly efficient photocatalytic overall water splitting. J. Colloid Interface Sci..

[cit44] Krysova H., Zlamalova M., Tarabkova H., Jirkovsky J., Frank O., Kohout M., Kavan L. (2019). Rutile TiO_2_ thin film electrodes with excellent blocking function and optical transparency. Electrochim. Acta.

[cit45] Cinar B., Kerimonglu I., Tonbul B., Demirbuken A., Dursun S., Kaya I. C., Kalem V., Akyildiz H. (2020). Hydrothermal/electrospinning synthesis of CuO plate-like particles/TiO_2_ fibers heterostructures for high-efficiency photocatalytic degradation of organic dyes and phenolic pollutants. Mater. Sci. Semicond. Process..

[cit46] Park S. M., Razzaq A., Park Y. H., Sorcar S., Park Y., Grimes C. A., In S. I. (2016). Hybrid Cu_*x*_O–TiO_2_ heterostructured composites for photocatalytic CO_2_ reduction into methane using solar irradiation: sunlight into fuel. ACS Omega.

[cit47] Bashiri R., Mohamed N. M., Kait C. F., Sufian S. (2015). Hydrogen production from water photosplitting using Cu/TiO_2_ nanoparticles: effect of hydrolysis rate and reaction medium. Int. J. Hydrogen Energy.

[cit48] Méndez-Medrano M. G., Kowalska E., Ohtani B., Uribe D. B., Colbeau-Justin C., Rau S., Rodríguez-López J. L., Remita H. (2020). Heterojunction
of CuO nanoclusters with TiO_2_ for photo-oxidation of organic compounds and for hydrogen production. J. Chem. Phys..

[cit49] Pham V. V., Bui D. P., Tran H. H., Cao M. T., Nguyen T. K., Kim Y. S., Le V. H. (2018). Photoreduction route for Cu_2_O/TiO_2_ nanotubes junction for encanced photocatalytic activity. RSC Adv..

[cit50] Mondal S., Reyes M. E. D. A., Pal U. (2017). Plasmon induced enhanced photocatalytic activity of gold loaded hydroxyapatite nanoparticles for methylene blue degradation under visible light. RSC Adv..

[cit51] Kumari R. M., Thapa N., Gupta N., Kumar A., Nimesh S. (2016). Antibacterial and photocatalytic degradation efficacy of silver nanoparticles biosynthesized using Cordia dichotoma leaf extract. Adv. Nat. Sci.: Nanosci. Nanotechnol..

[cit52] Rauf M. A., Meetani M. A., Khaleel A., Ahmed A. (2010). Photocatalytic degradation of methylene blue using a mixed catalyst and product analysis by LC/MS. Chem. Eng. J..

[cit53] Zhou S., Du Z., Li X., Zhang Y., He Y., Zhang Y. (2019). Degradation of methylene blue by natural manganese oxides: kinetics and transformation products. R. Soc. Open Sci..

[cit54] Marshall P. N. (1976). The composition of stains produced by the oxidation of methylene blue. Histochem. J..

[cit55] Pan G., Xu M., Zhou K., Meng Y., Chen H., Guo Y., Wu T. (2019). Photocatalytic degradation of methylene blue over layered double hydroxides using various divalent metal ions. Clays Clay Miner..

[cit56] Bahruji H., Bowker M., Davies P. R., Pedrono F. (2011). New insights into the mechanism of the photocatalytic reforming on Pd/TiO_2_. Appl. Catal., B.

[cit57] Guzman F., Chuang S. C., Yang C. (2013). Role of methanol sacrificing reagent in the photocatalytic evolution of hydrogen. Ind. Eng. Chem. Res..

